# Supplementary material for: Relation of Pulmonary Diffusing Capacity Decline to HRCT and VQ SPECT/CT Findings at Early Follow-Up after COVID-19: A Prospective Cohort Study (The SECURe Study)
Source: J Clin Med. 2022 Sep 26;11(19):5687. doi: 10.3390/jcm11195687 (PMC9572695; doi:10.3390/jcm11195687)
Supplement: Supplementary file 1 [file jcm-11-05687-s001.zip › jcm-1861703-supplementary.pdf]

Online supplemental files: 8

Methods:

Online supplemental file S1: Physical performance testing

Online supplemental file S2: Lung function testing

Online supplemental file S3: HRCT chest scan

Online supplemental file S4: VQ scintigraphy

Results:

Online supplemental file S5: Subjective complaints, physical performance and employment status

Online supplemental Table S1: Physical performance outcome 5 months after testing SARS CoV-2 positive (n=65) and difference between patients who were not hospitalised, hospitalised without ICU and hospitalised with ICU treatment

Online supplemental Table S2: Association between V/Q scintigraphy defects and HRCT findings of ground glass opacities (GGO) and sign of fibrosis (PF) in patients 5 months after testing SARS CoV-2 positive (n=65).

Online supplemental File S8: References.

Online supplemental file S1: Physical performance testing

Physical performance is assessed through Hand Grip strength (HGS) and 30-second Sit-To-Stand Test (STS) muscle strength tests and the Six-Minutes' Walk Test (6MWT). Maximal HGS is measured using a Jamar hand-held dynamometer and functions as a surrogate outcome measure for upper-body strength and assessment was done in accordance with the assessment protocol. STS was assessed as an outcome for lower limb strength and assessed in accordance with the test protocol [1].

Physical performance is assessed by the submaximal 6MWT walking distance (on a 30m track). Total walking distance, saturation, and degree of Perceived Exertion using the Borg Category-Ratio Scale (CR10) were obtained [2]. A walking distance during the 6MWT less than 357 meters is found to be a predicter for exacerbation-related hospital admission [3].

Online supplemental file S2: Lung function testing

Lung function testing included dynamic spirometry, body plethysmography and single breath measurement of DLco, in accordance with the ERS/ATS guidelines [4–6]. The tests were conducted at a dedicated lung function facility at the Department of Clinical Physiology and Nuclear Medicine at Rigshospitalet using a MasterScreen (Vyaire Medical, Würtzburg, Germany). Before measurements, standing height (to nearest 1 mm), weight (to nearest 100 g), and haemoglobin (Hb) (to nearest 0.1 mmol/L) of the participants were obtained. Hb was measured in capillary blood using HemoCue® (Hb 201+; HemoCue, Denmark). The variables reported in the present study are forced expiratory volume in the first second (FEV_1), forced expiratory volume (FVC), FEV_1/FVC-

ratio, total lung capacity (TLC), residual volume (RV), RV/TLC-ratio, Hb corrected DLco and diffusion coefficient for CO (Kco). These were reported as raw values and as % of predicted using ERS reference values for normalization for height, sex and age as appropriate. Participants in which FEV_1/FVC-ratio and TLC were below the lower limit of normal were classified as having obstructive and restrictive ventilation, respectively.

Online supplemental file S3: HRCT chest scan

The HRCT protocol consisted of two successive acquisitions with the patients scanned in the supine position after a breath-hold at deep inspiration and deep expiration, respectively. The following parameters were used: 130 kV, quality reference 120 mAs, cranio-caudal/ caudo-cranial direction, acquisition 16 x 1.2 mm, pitch 1.0, rotation time 0.6, and slice thickness 1.5 mm. Two experienced specialists (TKL (pulmonary medicine) & AK (radiologist)) read all HRCT chest scans in consensus. The readers divided each scan into six zones (three on each side), which were all evaluated for 1) ground glass opacities (GGO), 2) pulmonary fibrosis (PF), as indicated by the combination of reticulation, traction and bronchiectasis, in combination or separate, and 3) honeycombing (HC). For each of these findings, the extent in every zone was scored from 0 to 4: 0 = none (normal); 1 = 1-25%; 2 = 26-50%; 3 = 51-75%; 4 = > 75% of the zone [7]. Furthermore, the CT scans were evaluated for emphysema, nodules, tracheo- and/or bronchomalacia, bronchiectasis, and air-trapping.

Online supplemental file S4: VQ scintigraphy

VQ scintigraphy was conducted as a single photon emission computed tomography (SPECT) on a dual-head 16-slice CT Siemens Intevo BOLD SPECT/CT scanner (Siemens, Erlangen, Germany) by a simultaneous dual-isotope technique with ^{81m}Kr-gas as the ventilation tracer and ^{99m}Tc-macroaggregated albumin (MAA) as the perfusion tracer [8]. A 128*128 matrix, 128 angles, a MELP collimator and 11 min total acquisition time, as well as iterative Flash 3D reconstruction (3D OSEM 4 iterations, 16 subsets, 12 mm FWHM Guess filter, Siemens) were used. A low dose CT scan was obtained at free breathing (130 kV, Quality reference 30 mAs, acquisition 16 x 0.6, rotation time 0.6 sec, pitch 1.2, slice thickness 3 mm, cranio-caudal direction) for attenuation correction. Two experienced nuclear medicine specialists (JM & RB) read all VQ SPECT/CT scans in consensus. Interpretation criteria as recommended by European Association of Nuclear Medicine (EANM) were applied [9]. Hence, perfusion and ventilation defects were visually identified, localised, and classified as mismatched (only defect in perfusion), matched (combined perfusion and ventilation defect) or inversely mismatched (only defect in ventilation), and sized as subsegmental or segmental. A matched or inversely mismatched ventilation defect was classified as a ventilatory abnormality, regardless of concomitant HRCT findings, while a mismatched perfusion defect without any concomitant signs of fibrosis in the same area on HRCT, including reticulation with or without GGO, was classified as a vascular abnormality, most likely pulmonary embolism. However, if the HRCT showed signs of fibrosis precisely corresponding to a perfusion defect, it was interpreted as a ventilatory abnormality. Various studies have shown that interstitial lung fibrosis may cause mismatched perfusion defects that may incorrectly be interpreted as pulmonary embolism if not correlated to concomitant CT findings [10-13].

Online supplemental file S5: Subjective complaints, physical performance and employment status.

Most of the study participants reported at least one subjective complaint related to their COVID-19. Frequently reported symptoms included fatigue (68%), smell and taste disturbances (40%), weight changes, mainly weight loss (39%), concentration difficulties (37%), sleeping problems (29%), mood changes (20%) and excessive hair loss (19%). Only 7% (5/67) reported no COVID-19 related symptoms at the first follow-up visit. Of these two had not been hospitalised. The remaining three patients had been hospitalised for 1, 13 and 15 days, respectively, none of whom had required ICU admission. Likewise, many of the study participants reported ongoing respiratory challenges. The median (range) CAT score was 5 (IQR: 2-8).

In the response to the question "In general, how would you say your health is", with the possibilities to answer; excellent, very good, good, fair and poor, the distributions of answers to these categories were 12, 22, 35, 28 and 13%, respectively. We also asked the patients "compared to one year ago how would you rate your health in general now?", with the following answer options; much better, somewhat better, about the same, somewhat worse and much worse than one year ago. Here, the distributions of ratings to the five categories were 0, 6, 32, 47 and 15%. With regards to vigorous activities, such as running, lifting heavy objects, participating in strenuous sports 43, 38 and 18% of the study participants reported being limited a lot, a little or not limited at all.

Only five patients walked below the predictive cut-off for exacerbation-related hospital admission, however, those who did were all from the hospitalised groups (Supplemental Table 1). SpO_2 ob-

tained immediately after the 6MWT was significantly higher in the not hospitalised groups compared to both hospitalised groups. In general, lower limb strength was impaired for all three groups when compared to age- and sex adjusted reference norms (71%), however upper-body strength was not comparable impaired when compared to age- and sex adjusted reference norms (93%).

At the follow-up visit data for 65 study participants regarding employment status was available; 36 (55%) had resumed work, while 10 and 2 (15% and 3%) were still on partly or full sick leave. Thirteen patients (20%) were retired while 4 patients (8%) did not fit to any of the above categories. Translating into 75% (36/48) of those working prior to the COVID-19 illness having resumed full-time work at follow-up.

Online Supplemental Table S1. Physical performance outcome median 5 months after testing SARS CoV-2 positive (n=65) and difference between patients who were not hospitalised, hospitalised without ICU and hospitalised with ICU treatment

	**All (n=65)**	**Group I (n=12)**	**Group II (n=39)**	**Group III (n=14)**	**P-value (between groups)#**
**Before testing**					
GRS†	6 [4;8]	7 [5.5;8.8]	6 [4;8]***	5 [4;6]*	0.280
SpO2 at rest**	98 [96;99]	99 [99;99]*	98 [96;98]*	97 [96;98]	0.002[A]
CR10 at rest§	0.0 [0.0;0.7]	0.0 [0.0;0.7]	0.0 [0.0;0.9]***	0.2 [0.0;0.6]**	0.732
**Hand Grip Strength**					
HGS, Kg	38.3 ± 11.6	36.3 ± 8.7	40.5 ± 11.1	33.7 ± 13.8	0.128
HGS, %norm	93.0 [77.5;104.1]	93.3 [90.5;98.3]	100.8 [77.9;112.2]	72.6 [63.7;92.1]	0.018[B]
**Sit-To-Stand**					
STS, repetitions**	15 [13;20]	18.5 [15.5;25.5]	14 [12;18]*	14 [11;20]*	0.046[C]
STS, % norm**	68.6 [55.8;85.8]	71.8 [58.8;106.2]	67.8 [55.8;82.3]*	66.9 [60.1;85.8]*	0.547
SpO2 after STS¤	97 [96;98]	99 [97;100] €	97 [96;98] °	97 [95;97]***	0.059
CR10 after STS §	3.0 [2.0;4.5]	2.5 [1.0;3.0]*	2.5 [2.0;4.0]***	4.0 [3.0;6.0]*	0.051
**6 Minute Walking Test**					

6MWT, walked meters	548 ± 135	648 ± 122	529 ± 135	516 ± 115	0.015[A]
6MWT, %norm	94.3 [88.1;104.8]	96.1 [89.2;107.9]	95.0 [86.9;107.3]	90.3 [84.6;97.8]	0.575
6MWT below 357m	5 (7.7)	0 (0.0)	4 (10.3)	1 (7.1)	0.814
SpO2 after 6MWT φ	97 [96;99]	99 [98;100] †	97 [96;98] Δ	97 [95;97] £	0.036[D]
CR10 after 6MWT †	3.0 [2.0;5.0]	2.0 [1.0;4.0]	3.0 [2.0;5.0]*	4.0 [3.0;7.0]***	0.049[D]

Data are expressed as mean ± SD, median [interquartile range] or n(%).

GRS = Global Rating Scale for overall health status, SpO2 = Partial Oxygen Saturation, CR10 Borg = Borg Category-Ratio scale, Hand Grip Strength = Maximal Hand Grip Strength, % norm = percent of population norm adjusted for sex and age, STS = Sit-To-Stand Repetitions during 30 second sit to stand, 6MWT = 6-Minute Walking Test,

* Data missing from one patient, ** Data missing from two patients, *** Data missing from three patients,

† Data missing from four patients, § Data missing from five patients, € Data missing from six patients, £ Data missing from seven patients, Δ Data missing from eight patients, ° Data missing from nine patients

¤ Data missing from 18 patients, φ Data missing from 19 patients

One-way ANOVA or Kruskal-Wallis equality-of-populations rank test (with ties) where appropriate. If significant followed by bivariate comparison using Dunn's test with Bonferroni correction for multiple comparisons

A: Difference between not hospitalised and hospitalised without ICU, and not hospitalised and hospitalised with ICU

B: Difference between hospitalised without ICU and hospitalised with ICU

C: Difference between not hospitalised and hospitalised without ICU

D: Difference between not hospitalised and hospitalised with ICU

Data are expressed as median and interquartile range (IQR).
GRS = Global Rating Scale for overall health status, SpO2 = Partial Oxygen Saturation, CR10 Borg = Borg Category-Ratio scale, Hand Grip Strength = Maximal Hand Grip Strength, % norm = percent of population norm adjusted for sex and age, STS = Sit-To-Stand Repetitions during 30 second sit to stand, 6MWT = 6-Minute Walking Test, *Kruskal-Wallis equality-of-populations rank test (with ties)

Online Supplemental Table S2: Association between V/Q scintigraphy defects and HRCT findings of ground glass opacities (GGO) and sign of fibrosis (PF) in patients 5 months after testing SARS CoV-2 positive (n=65). Data expressed as n. * Data missing from one patient (n=64); #Fisher's exact test

	**Mismatched Q defects**		**P-values#**
	Yes	No	
GGO on HRCT*			
>25%	13	4	0.386
≤25%	30	17	
PF on HRCT*			
Yes	20	8	0.598
No	23	13	
Matched V/Q defects			
Yes	19	7	0.426
No	24	15	

	Matched V/Q defects		P-values#
	Yes	No	
GGO on HRCT*			
>25%	8	9	0.563
≤25%	17	30	
PF on HRCT*			
Yes	14	14	0.130
No	11	25	

	GGO on HRCT		P-values#
	>25%	≤25%	
PF on HRCT*			
Yes	16	12	<0.001
No	1	35	

	Any V/Q defects		P-values#
	Yes	No	
GGO (>25%) or PF on HRCT*			
Yes	28	1	1.000
No	33	2	

References.

1. Csuka, M.; McCarty, D.J. Simple Method for Measurement of Lower Extremity Muscle Strength. *Am. J. Med.* **1985**, *78*, 77–81. https://doi.org/10.1016/0002-9343(85)90465-6.
2. Borg, G. *Borg's Perceived Exertion and Pain Scales*; Human kinetics: Champaign, IL, USA, 1998; pp. 13–16.
3. Spruit, M.A.; Polkey, M.I.; Celli, B.; Edwards, L.D.; Watkins, M.L.; Pinto-Plata, V.; Vestbo, J.; Calverley, P.M.A.; Tal-Singer, R.; Agusti, A.; et al. Predicting Outcomes from 6-Minute Walk Distance in Chronic Obstructive Pulmonary Disease. *J. Am. Med. Dir. Assoc.* **2012**, *13*, 291–297. https://doi.org/10.1016/j.jamda.2011.06.009.
4. Miller, M.R.; Hankinson, J.; Brusasco, V.; Burgos, F.; Casaburi, R.; Coates, A.; Crapo, R.; Enright, P.; van der Grinten, C.P.M.; Gustafsson, P.; et al. Standardisation of Spirometry. *Eur. Respir. J.* **2005**, *26*, 319–338. https://doi.org/10.1183/09031936.05.00034805.
5. Wanger, J.; Clausen, J.L.; Coates, A.; Pedersen, O.F.; Brusasco, V.; Burgos, F.; Casaburi, R.; Crapo, R.; Enright, P.; van der Grinten, C.P.M.; et al. Standardisation of the Measurement of Lung Volumes. *Eur. Respir. J.* **2005**, *26*, 511–522. https://doi.org/10.1183/09031936.05.00035005.
6. MacIntyre, N.; Crapo, R.O.; Viegi, G.; Johnson, D.C.; van der Grinten, C.P.M.; Brusasco, V.; Burgos, F.; Casaburi, R.; Coates, A.; Enright, P.; et al. Standardisation of the Single-Breath Determination of Carbon Monoxide Uptake in the Lung. *Eur. Respir. J.* **2005**, *26*, 720–735. https://doi.org/10.1183/09031936.05.00034905.
7. Goldin, J.G.; Lynch, D.A.; Strollo, D.C.; Suh, R.D.; Schraufnagel, D.E.; Clements, P.J.; Elashoff, R.M.; Furst, D.E.; Vasunilashorn, S.; McNitt-Gray, M.F.; et al. High-Resolution CT Scan Findings in Patients with Symptomatic Scleroderma-Related Interstitial Lung Disease. *Chest* **2008**, *134*, 358–367. https://doi.org/10.1378/CHEST.07-2444.
8. Mortensen, J.; Gutte, H. SPECT/CT and Pulmonary Embolism. *Eur. J. Nucl. Med. Mol. Imaging* **2014**, *41* (Suppl. 1), S81–S90. https://doi.org/10.1007/s00259-013-2614-5.
9. Bajc, M.; Schümichen, C.; Grüning, T.; Lindqvist, A.; Le Roux, P.-Y.; Alatri, A.; Bauer, R.W.; Dilic, M.; Neilly, B.; Verberne, H.J.; et al. EANM Guideline for Ventilation/Perfusion Single-Photon Emission Computed Tomography (SPECT) for Diagnosis of Pulmonary Embolism and Beyond. *Eur. J. Nucl. Med. Mol. Imaging* **2019**, *46*, 2429–2451. https://doi.org/10.1007/S00259-019-04450-0.

10. Strickland, N.H.; Hughes, J.M.B.; Hart, D.A.; Myers, M.J.; Lavender, J.P. Cause of Regional Ventilation-Perfusion Mismatching in Patients with Idiopathic Pulmonary Fibrosis: A Combined CT and Scintigraphic Study. *Am. J. Roentgenol.* **1993**, *161*, 719–725. https://doi.org/10.2214/ajr.161.4.8372745.
11. Gutte, H.; Mortensen, J.; Jensen, C.V.; Johnbeck, C.B.; Von Der Recke, P.; Petersen, C.L.; Kjærgaard, J.; Kristoffersen, U.S.; Kjær, A. Detection of Pulmonary Embolism with Combined Ventilation-Perfusion SPECT and Low-Dose CT: Head-to-Head Comparison with Multidetector CT Angiography. *J. Nucl. Med.* **2009**, *50*, 1987–1992. https://doi.org/10.2967/jnumed.108.061606.
12. Li, D.K.; Seltzer, S.E.; McNeil, B.J. V/Q Mismatches Unassociated with Pulmonary Embolism: Case Report and Review of the Literature. *J. Nucl. Med.* **1978**, *19*, 1331–1333.
13. Hughes, J.M.B.; Hart, D.; Carr, D.; Lavender, J.P.; Pride, N.B. Regional ventilation-perfusion mismatch in lung fibrosis: A false positive pulmonary embolism scan. *Thorax* **1987**, *42*, 229.
